# Editorial: Protein Interaction Networks in Health and Disease

**DOI:** 10.3389/fgene.2016.00111

**Published:** 2016-06-14

**Authors:** Spyros Petrakis, Miguel A. Andrade-Navarro

**Affiliations:** ^1^Centre for Research and Technology Hellas, Institute of Applied BiosciencesThessaloniki, Greece; ^2^Faculty of Biology, Johannes-Gutenberg UniversityMainz, Germany; ^3^Institute of Molecular BiologyMainz, Germany

**Keywords:** protein interactions, protein function, protein network, disease, systems biology

The identification and annotation of protein-protein interactions (PPIs) is of great importance in systems biology. Big data produced from experimental or computational approaches allow not only the construction of large protein interaction maps but also expand our knowledge on how proteins build up molecular complexes to perform sophisticated tasks inside a cell. However, if we want to accurately understand the functionality of these complexes, we need to go beyond the simple identification of PPIs. We need to know when and where an interaction happens in the cell and also understand the flow of information through a protein interaction network.

Another perspective of the research on PPI networks is the study of their relation to disease. In disease conditions, mutations that alter the secondary structure of one protein might perturb its PPIs, as well. Thereafter many things can go wrong via cascading effects, caused by the inter-relatedness of the mutated protein to other proteins through the PPI network. Such perturbations could block the formation of a protein complex or lead to the formation of new protein complexes and the activation of abnormal signaling pathways. These events could alter the cellular transcriptome profile and further contribute to disease pathogenesis. That is why the maintenance of the proper structure and functionality of a PPI network is crucial for cellular homeostasis. Its disruption can cause complex effects and understanding them requires advanced methods for analysis.

The aim of this Research Topic is to present novel findings and recent achievements in the field of PPI networks. Thematically, it is divided into two parts. First, we present methods for the identification and quantification of PPIs; second, we describe computational approaches to annotate interactomes and extract information related to disease prediction or disease progression.

The first four articles deal with the identification and quantification of PPIs. In the first work, Suter et al. describe the application of next generation sequencing (NGS) for the characterization of binary PPIs. Authors present an accurate method to analyze yeast two-hybrid data by NGS and also interpret interaction data via quantitative statistics. They also discuss how this methodology can be used to discover differential PPIs allowing the identification of disease mechanisms (Suter et al.).

The next two review articles describe mass spectrometry (MS) based approaches. Yang et al. present methods that can determine the relative abundance of purified proteins in a sample enabling the identification of transient PPIs in different conditions. Additionally, when combined with proximity tagging methods, MS may illuminate spatial or temporal PPIs, especially those of signaling pathways whose perturbation may underlie human diseases (Yang et al.). Meyer and Selbach indicate how MS can be used to identify dynamic changes in the interactome. Stable isotope labeling in aminoacids and affinity purification-MS can shed light on the dynamic behavior of proteins even at different stages of an experiment following perturbation. Authors also describe how MS may identify the stoichiometry of proteins in complexes. These methodologies can be employed to study the dynamic changes of PPIs under normal and disease conditions (Meyer and Selbach).

In the next article, Buntru et al. review novel cell-based assays for the detection of PPIs and discuss their strengths and weaknesses. Compared to traditional genetic or biochemical methods, these techniques provide quantitative information of PPIs even in the context of living cells. This information can be used to prioritize a large number of PPIs, allowing researchers to better describe the biological systems and improve our understanding of disease processes (Buntru et al.).

The second part of the Research Topic is comprised of seven papers dealing with the annotation of protein interaction networks. Alanis-Lobato describes computational mining tools to improve the reliability of protein networks and predict new interactions based on the topological characteristics of their components. He also provides examples on how the integration of clinical data can highlight disease modules in these networks or indicate similarities between diseases (Alanis-Lobato).

Pelassa and Fiumara study the functional role of homopolymeric amino acid repeats (AARs) in proteins and their PPIs. AARs are considered to mediate PPIs and in some cases correlate with human diseases, such as polyglutamine expansions involved in Huntington's disease. The authors describe a computational screening of the human interactome and show that AAR-containing network components have a high degree of connectivity. They also indicate an overlap between AARs and interaction domains suggesting that AARs play an important role in shaping protein interaction networks (Pelassa and Fiumara).

Lecca and Re present WG-Cluster, a novel algorithm for the detection of modular structures in protein networks. This tool combines network node and edge weight information of connected proteins improving the biological interpretability of a PPI. The authors also apply their technique in biological datasets from patients with colorectal cancer and indicate differentially active cellular processes in normal vs. tumor conditions (Lecca and Re).

In the next article, Chen and colleagues use the dynamical network biomarkers method to detect early disease signals in a breast cancer cell model. The authors pinpoint critical network changes and highlight a number of pathways associated with the pre-transition from the normal state to a cancer cell progression stage. They also suggest the use of these signals as targets for disease intervention (Chen et al.).

Databases collecting data on experimentally verified PPIs are a valuable resource for the research community. In particular, there are studies that extract biological knowledge from analysis of these global data. However, the different intensity with which different proteins have been studied, influences the amount of data that is available for certain proteins leading to wrong statements. Schaefer et al. study the biases that affect the human PPI data due to research heterogeneity, propose measures to correct this and show an application to proteins involved in cancer.

Yeger-Lotem and Sharan present computational approaches to construct tissue or disease-specific interactomes. They indicate how the combination of transcriptome profiles with proteomics data could categorize PPIs from large networks according to their occurrence in specific tissues. In parallel, they present the effect of disease-causing mutations on protein stability and subsequently on the integrity and structure of protein networks in an affected tissue (Yeger-Lotem and Sharan).

In the last article of this topic, Theofilatos et al. argue about the challenges of computational analysis of PPI data and present future goals such as biomarker discovery or identification of pathogenic PPIs and their drug targeting. Authors also support that the integration of environmental or clinical data in protein networks will allow their in-depth study and the construction of personalized interactomes (Theofilatos et al.).

In the past decade, network biology focused on the representation of the binary interaction of proteins. Today, the field of PPI research capitalizes and hops above the establishment of such previous work and resources, identifies existing limitations, and proposes further avenues of investigation, as reflected in this Frontiers Research Topic. A tight connection between experimental and computational efforts is a hallmark of the articles that we present here, which set the tone that PPI research will follow in the next years. If anything remains unchanged, this is our awareness of the fact that diseases are often caused by the malfunction of large protein complexes. This holds as the main motivation of research in the field, which screams for more complete and reliable interactomes, ultimately crucial in order to identify relevant pathogenic mechanisms and design therapeutic intervention strategies.

## Author contributions

All authors listed, have made substantial, direct and intellectual contribution to the work, and approved it for publication.

### Conflict of interest statement

The authors declare that the research was conducted in the absence of any commercial or financial relationships that could be construed as a potential conflict of interest.

